# Animal learning may contribute to both problems and solutions for wildlife–train collisions

**DOI:** 10.1098/rstb.2018.0050

**Published:** 2019-07-29

**Authors:** Colleen Cassady St. Clair, Jonathan Backs, Alyssa Friesen, Aditya Gangadharan, Patrick Gilhooly, Maureen Murray, Sonya Pollock

**Affiliations:** Department of Biological Sciences, University of Alberta, Edmonton, Canada T6G 2E9

**Keywords:** transportation, railway, mitigation, ecological trap, behavioural flexibility, grizzly bears

## Abstract

Transportation infrastructure can cause an ecological trap if it attracts wildlife for foraging and travel opportunities, while increasing the risk of mortality from collisions. This situation occurs for a vulnerable population of grizzly bears (*Ursus arctos*) in Banff National Park, Canada, where train strikes have become a leading cause of mortality. We explored this problem with analyses of rail-associated food attractants, habitat use of GPS-collared bears and patterns of past mortality. Bears appeared to be attracted to grain spilled from rail cars, enhanced growth of adjacent vegetation and train-killed ungulates with rail use that increased in spring and autumn, and in areas where trains slowed, topography was rugged, and human density was low. However, areas with higher grain deposits or greater use by bears did not predict sites of past mortality. The onset of reported train strikes occurred amid several other interacting changes in this landscape, including the cessation of lethal bear management, changes in the distribution and abundance of ungulates, increasing human use and new anthropogenic features. We posit that rapid learning by bears is critical to their persistence in this landscape and that this capacity might be enhanced to prevent train strikes in future with simple warning devices, such as the one we invented, that signal approaching trains.

This article is part of the theme issue ‘Linking behaviour to dynamics of populations and communities: application of novel approaches in behavioural ecology to conservation’.

## 1. Introduction

Road and railway networks are among the human infrastructure that can attract wildlife with apparent benefits, while imposing net costs to individuals, to cause population declines known as ecological traps [1,2]. Wildlife may be attracted to transportation features by foraging opportunities and movement efficiency, which generate net benefits for many species [3,4]. For other species, transportation features exert mainly negative effects, which include habitat loss, degradation and fragmentation, as well as mortality from collisions with vehicles [5–9]. Species that avoid roads are less likely to experience ecological traps, but their populations are more likely to decline owing to habitat loss [10] and reduced genetic exchange [11] that further reduces population viability [12]. The complexity of identifying and appropriately mitigating the multiple effects of transportation features on wildlife poses an immense challenge for managers, making these topics a perennial focus of conservation biology and the sub-discipline of road ecology.

Decades of work on road mitigation revealed a gold standard that consists of wildlife fencing to prevent collisions combined with periodic crossing structures to support wildlife movement [12,13]. Some also advocate this form of mitigation for railways [14]. This approach is especially effective in protected areas that contain sensitive or declining populations [15], which often include carnivores [16]. In addition to low population densities and large home ranges, carnivores are typified by high behavioural flexibility that may increase their susceptibility to ecological traps [17], including those imposed by roads [18,19]. Carnivores, and especially grizzly bears (*Ursus arctos*), were among the species targeted by road mitigation that occurred in Banff National Park, Canada [20]. Unfortunately, no similar mitigation occurred on an adjacent railway, which became the leading local cause of mortality for this species [21].

The imbalance in road and railway mitigation in Banff is not unique. Only a few studies have examined the effects of railways on wildlife [7,9,22] and almost none have attempted to mitigate it [23]. One reason for this neglect is the lacking incentive for protecting human lives, but another is the much lower frequency of trains, relative to vehicles on roads, which logically (but not always) reduces each of mortality, avoidance, and the benefit of expensive mitigation with exclusion fencing and crossing structures [7]. These limitations are especially likely in mountainous areas, where railways often occupy the most productive and accessible habitat for wildlife. Nonetheless, growing evidence shows that wildlife–train collisions substantially impact some populations, particularly when railways provide food that attracts wildlife [24] or when they traverse wilderness areas containing wide-ranging species [25,26]. These negative impacts have been documented for several other populations of grizzly or brown bears [27–29] for which rail-caused mortality sometimes exceeds that of roads [27,29].

The challenges for effective railway mitigation might contribute to solutions if they were viewed differently through the mechanistic lens of an ecological trap, which results not from the human infrastructure *per se*, but from the behavioural processes of individuals via cue recognition, perception, experience and adaptation [2,30,31]. If an animal misjudges cue meaning or resource value, both partially functions of experience, it is more likely to exhibit maladaptive behaviours. It follows that experienced animals with sufficient behavioural flexibility could learn to avoid the detrimental effects of roads or railways, even without mitigation that prevents wildlife access. Carnivores may be especially likely to do this because they are capable of rapid adaptation to new anthropogenic features [32,33] and exhibit behavioural responses to roads that accommodate changing traffic volume [34], types of crossing structures [35] and locations for specific activities [36].

This combined context supported our study in Banff and Yoho National Parks, Canada, with goals to understand the root causes of grizzly bear vulnerability to train mortality and recommend effective forms of mitigation. We formed one of several research teams that was funded and logistically supported by the Joint Initiative for Grizzly Bear Conservation formed by Canadian Pacific Ltd. and Parks Canada Agency (hereafter PCA) [37]. That initiative sprang from the recent threatened designation for the provincial population of grizzly bears [38], evidence of local population decline [39], public debate about how best to protect the grizzly bears of Banff [40] and popular media that attributed the mortality largely to grain spilled on the railway [41]. In the sections below, we synthesize our research contributions to that initiative, show why there is no simple environmental explanation for bear vulnerability to train strikes, emphasize the necessity of individual learning in this rapidly changing environment, and describe a warning device with which managers might accelerate learning by wildlife to detect approaching trains, thus preventing population-level ecological traps.

## 2. Study area and methods

Our work was based in Banff National Park, Alberta, and extended through Yoho National Park, British Columbia, both of which are bisected by the Canadian Pacific (hereafter CP) mainline and comprise part of the Canadian Rocky Mountain Parks, a UNESCO World Heritage Site. Grizzly bears in the study area persist at unusually low population densities that stem partly from a harsh environment with low vegetative productivity [42] and high rates of human-associated mortality [43].

This railway was completed in 1885, the same year that Banff became Canada's first national park, with a primary, ongoing purpose to transport cargo to and from the port of Vancouver. Westbound goods include grain and other agricultural products from the prairies that are transported in hopper cars, which are loaded from the top and emptied into shipping containers from the bottom. Faulty gates cause grain to leak from hopper cars, which is consumed by bears [44] and appears to increase the time they spend on the railway searching for spilled grain [41]. Railway mortality of other wildlife species has been documented since 1982, with reporting stringency that increased in 1996, and the first report of a grizzly bear strike in 2000. Since then, site inspections confirmed train-caused mortalities of 14 grizzly bears resulting from 11 reported events (Government of Canada Open Data). An additional seven bears in six strike events were reported by train crews, but could not be confirmed as mortalities via the presence of bear carcasses.

The work described below used data and information collected by PCA employees, both during the project and over the past few decades. These sources included several Geographic Information System layers describing natural features (e.g. vegetation, topography, elevation, water bodies), remotely sensed variables (e.g. vegetation greenness), and anthropogenic features related to accommodation (e.g. towns, campgrounds, resorts), transportation (e.g. roads, railway, gravel pits), recreation (e.g. trails and ski hills) and other categories (e.g. power lines and fence lines). We made repeated use of a database describing date, time, location and identifying information for all mortalities recorded in the park, including those associated with transportation (both roads and rail) for animals as small as coyotes.

Each spring between 2012 and 2015, PCA employees captured and fitted global positioning system (GPS) collars on grizzly bears inhabiting the front country of Banff and Yoho National Parks. We used resulting data to study how bears interacted with the railway and other features, both natural and anthropogenic, in the study area. Detailed methods for the results described below are contained in the associated papers.

## 3. Synthesis of results

A founding rationale for our study was to test the hypothesis that grain spilled from hopper cars was responsible for increasing rates of grizzly bear mortality. To support this goal, we developed and refined a method to quantify spilled products that could be compared over time and among locations. As an ecological input, annual rates of grain spillage were substantial, summing to an estimated total in our study area of 110 tonnes annually, but represented a tiny fraction of the grain that was shipped [45]. Grain deposition was best predicted by shipping rates, which increase each autumn and winter, and were higher where trains stopped or travelled slowly, consistent with the effects of slow leaks [45].

In addition to grain, we found evidence that the railway supplements grizzly bear diet in the form of enhanced vegetation growth, comparable to what occurs for roads [6]. We measured this effect using repeated vegetation surveys on paired transects on the edge of the rail bed (i.e. ballast), forest edge, and within the forest at sites used for grain sampling. Both diversity (alpha and beta) and abundance (per cent cover and berry production) of plants palatable to bears were higher on the forest edge where there was up to a fivefold increase in berry production for some species [46]. The positive effect of forest edge on berry abundance and ripening increased with elevation [46]. Vegetation enhancement on the rail was generally lower than for other linear features in this area, particularly in comparison to powerlines in summer [47]. Relative to reference samples, vegetation growing near the rail and, especially, spilled grain contained higher concentrations of heavy metals and polycyclic aromatic hydrocarbons, which are potentially associated with diesel emissions from train engines, material abrasion from wheels, lubrication products and creosote-treated railway ties [48].

To identify and synthesize information about attractants that encourage rail use, we used analyses of stable isotopes in bear hair collected during capture, locations from GPS-collared bears, and scats collected both opportunistically and at sites of known bear use. Relative to bears from other sites, we found elevated metabolism of nitrogen and sulfur in rail-associated bears [49], but these did not correlate quantitatively with the amount of rail use by individual bears, which was highly variable among 21 collared individuals [50]. Only four bears used the rail more often than 10% of the days they were monitored and each of these produced scat containing grain, particularly in autumn [50]. Almost half of all scats found near the rail contained grain, but these scats also had higher diversity of food types and were more likely to contain ungulate and ant remains in summer [50].

Bears that made frequent use of the rail included a large, dominant male bear whose rail use was concentrated where rates of ungulate mortality were high, and three small sub-adults [50] that might have been especially likely to seek food opportunistically. One of these sub-adult bears exhibited aggressive defence of a hole in the ground that contained cached grain. This observation caused us to explore the possibility that red squirrels (*Tamiasciurus hudsonicus*) living adjacent to the rail cached grain that was later excavated by bears, potentially conditioning them to seek grain on the rail. As predicted, squirrels occurred at higher densities near the rail where we also found middens containing agricultural products [51]. We also saw (via remote camera) a young study bear excavate a midden containing grain and found evidence of digging at several others.

We examined correlates of rail mortality more generally by determining whether it increased after mitigation of the adjacent highway through Banff National Park, which occurred in discrete sections between 1983 and 2013. This work confirmed an earlier assessment that road mortality declined dramatically after mitigation [20], but it also showed that mortality of the most abundant species, elk (*Cervus canadensis*), was more dependent on population size than mitigation [52]. After highway mitigation, rail mortality increased on the railway for other ungulates, but also over time, suggesting that it might have been driven partly by increasing population sizes for deer (*Odocoileus* spp.). Highway mitigation had no measurable effect on carnivore mortality on either the road or railway, but bear mortality increased slightly through time [52].

Regardless of which attractant or human activity incents bears to use the railway, it could increase strike risk either numerically, with time spent, or functionally via encounter context. We explored these mechanisms with GPS-collared bears via overall habitat use near the rail and locations of four types of steps defined by three successive locations that entered, continued, exited, or crossed the rail [53]. Bears were more likely to continue (via three successive points on the rail) where trains travelled slowly, where topography was most rugged, and away from high human density. Slower trains with leaking cars deposit more grain [45], especially at railway sidings where trains stop while others pass, and heightened rail use in rugged terrain logically increases travel efficiency for bears. Surprisingly, continue sites were *negatively* associated with the frequency of past mortality, whereas other movement types and overall rail were not correlated with mortality. This finding suggests that higher encounter rates between bears and trains at locations with more spilled grain may not produce greater strike risk because of slower train speeds, consistent with earlier work by others showing a positive relationship between bear strikes and train speed [44].

Our empirical work did not find much evidence to support a prevalent hypothesis at the beginning of our study that grain deposits caused grizzly bear strikes. Among the sites we used to monitor grain (above), nine associated with past strikes (confirmed and unconfirmed; Fig. 1 in [45]) exhibited average accumulations of agricultural products combined (*x* = 7.5 ± 2.4 g m^−2^ week^−1^) that were half as large as accumulations at nine sites that were not associated with previous strikes (*x* = 15.7 ± 4.9 g m^−2^ week^−1^). Moreover, bear strikes were most common in May and June (9/17 events) when grain deposition rates were relatively low [45]. However, past spillage rates may have been higher with greater tendencies to contribute to collisions. Among all 17 collision events with grizzly bears (confirmed and reported), all but four (one confirmed mortality of two cubs and three reported strikes) occurred before our study began in 2012 and after the retrofit of thousands of hopper cars (2007–2011) to repair leaky gates [54].

Several other spatial and temporal factors potentially contribute to bear–train collisions, but untangling their effects was difficult with just 11 confirmed mortality sites and is the subject of ongoing modelling work. We were especially interested in two hotspots of confirmed grizzly bear mortality near the townsites of Banff and Lake Louise [53]. Both hotspots occur near highway overpasses and interchanges with a secondary road where curving track occurs immediately beside the Bow River or its tributaries. These complex features increase the likelihood that bears fail to detect or escape approaching trains, but they cannot alone explain the sudden onset of train-caused mortality. We wondered what else had changed in this landscape over the past few decades with relevance to bear mortality. We speculate about those changes below in the spirit of a prophesy voiced by a PCA official who launched the research initiative in 2011: just as we found no silver bullets for potential mitigation, we [tried to ensure we] overlooked no sacred cows of potential causation.

## 4. Bear survival in a changing landscape requires rapid learning

Public concern about bears being struck by trains while searching for grain on the railway [21] may have overestimated the magnitude of the mortality increase while underestimating the relevance of several other landscape-level changes. In the reporting period (1982–2017) of the mortality database we used above, one profound change for grizzly bears was clear; lethal management and translocations effectively ceased. Dividing the mortality database into two 17-year periods before the onset of train-caused mortality reports for grizzly bears (1982–1999; *n* = 42) and after (2000–2017; *n* = 49) revealed a modest 17% increase in the second period, but very different causes. Almost all losses resulting from management action (lethal management and translocations combined) occurred in the first period (96% of 24), whereas most mortality attributed to collisions (roads and rail combined) occurred in the second one (84% of 32). More stringent reporting requirements on the railway after 1996 may have amplified this difference, but there is no doubt that a paradigm shift in bear management occurred in the years before train collisions began. This transition began in the 1980s in Banff [43] and elsewhere [55] as park users improved securement of food attractants to avoid conflict with bears [56], while habituated bears learned greater wariness via hazing and aversive conditioning from managers [57].

The new paradigm for bear management emerged amid a suite of other ecological and anthropogenic changes that likely encouraged bear use of the railway, particularly near townsites, but with complexity that would be hard to anticipate. One of these changes was recolonization of the area by wolves (*Canis lupus*) beginning in about 1985 [58] at the peak of the local, hyper-abundant elk population (*Cervus canadensis*), which began to decline shortly thereafter (Fig. 3 in [52]). Wolves increased the tendency for elk to congregate near the Banff townsite as a predator refuge [59] where they damaged deciduous ecosystems [60] and threatened the safety of visitors [61] that were increasing at a rate of 5.5% annually [62]. These resident elk also attracted carnivores [63], including grizzly bears that are especially dependent on elk calves [64,65]. Managers attempted to disperse the resident elk population, which peaked at many hundred individuals [52], with habitat restoration for carnivores [66] and elk management that included translocations, exclusion barriers and removal of the most habituated individuals. These actions may have contributed to the tendency for elk to use the railway to access the townsite, further concentrating elk–train collisions that peaked in the year 2000 (Fig. 3 in [52]), the same year that reports of collisions with grizzly bears began.

By 2002, managers and wolves had succeeded in reducing the size of the elk population by 65% [52], just as a comparative study of grizzly bears revealed the local population to have unusually low reproductive rates that were likely related to protein limitation [42]. Grizzly bears may be further limited by berry crops that have declined because of forest succession, fire suppression and climate change [67]. Food limitation supports the logic of collecting carcasses generated by collisions and management actions, and redistributing them in the landscape to be scavenged by carnivores. A similar practice, known as intercept feeding, can reduce carnivore mortality stemming from human–wildlife conflict [68]. In Banff, these carcasses were sometimes placed at road-accessible sites and one of these sites was beside the Bow River, opposite the railway zone that would become a mortality hotspot for grizzly bears. This site attracted high visitation rates by multiple GPS-collared bears during our study. More recently, carcasses have been divided into smaller portions and distributed more widely.

With the benefit of hindsight, one can readily imagine how food-limited bears would learn to explore and then exploit sites with predictable ungulate carcasses. Elsewhere, grizzly bears exhibit extensive and long-lasting attraction to carcass pits for livestock [69], which they can detect by olfaction over many kilometres [70]. Similar carcass pits attract carnivores around the world, often with profound, but underappreciated, ecological effects [24]. Profitable foraging sites tend to attract repeated visits by bears partly owing to keen memories and the capacity for cultural transmission [71]. These abilities may have contributed to the tendency of our study bears to revisit old gravel pits that offer herbaceous forage, but likely also retain scent cues from their history as landfills that closed decades ago [43]. In addition to point sources of attraction, changes in the composition of the ungulate community may encourage bears to search the rail extensively in search of dispersed carcasses. These would result from increasing populations of white-tailed deer (*Odocoileus virginianus*) that are especially attracted to train-spilled cereal grains and canola [72], and moose (*Alces alces*) that thrive in wetter areas. This search strategy seemed especially apparent in our largest study bear (hereafter, M122).

M122 and other study bears provided anecdotal evidence about how bears interact with the rail that is hard to quantify, but nonetheless suggest that learning might prevent enough wildlife–train collisions to avoid a population-level ecological trap. M122 gained mass partly by targeting carcasses along the railway; large size presumably supported his dominance over other bears and ability to sire a majority of recent cubs in the population (D. Garrow 2015, personal communication). These gains may have been possible because he was reportedly grazed by a train, perhaps making him especially attentive to detecting and evading them. The effect of vigilance is evoked by a 2013 photograph of two sub-adult male bears. The older one (M126) seemed shier, later featured in a media story by fleeing from an approaching train straight up a steep hill, and survived to the end of our study. The younger one (M128) had been orphaned by a previous train strike, was reported for conflict with people, and was later killed in a vehicle collision on a nearby highway. An adult female (F130) that seldom used the rail lost two cubs to a train collision in 2012, but led two more cubs to almost exactly the same location 2 years later. They appeared to be struck by a train, but were still alive the following spring. Had the female gained enough experience to huff a warning that prevented a collision with seconds to spare? Among eight (of 28) study bears that died by the end of 2017, four sub-adults and one young adult died from anthropogenic causes, whereas three elderly females apparently died of natural causes. Inexperience of perception or reaction is presumably the reason that young animals are more prevalent in wildlife–vehicle collisions for grizzly bears [28,29] and many other species [73].

## 5. Learning-based rail mitigation

Although our appreciation for the importance of learning by grizzly bears grew during our study, we anticipated its relevance from the beginning, recognizing how its benefit to individuals could scale up to the population. Consequently, we imagined a warning system to alert bears (and other wildlife) of approaching trains. We reasoned that bears could obtain important benefits of rail use, with less risk of mortality, if they could better detect and avoid trains. Importantly, our goal was not to prevent rail access or to eliminate mortality completely. A European population of brown bears with much higher annual mortality from train collisions still exhibits population growth [74]. Rather, we imagined that the ability to recognize and avoid trains might be especially important in some contexts, such as if it occurred at hotspots of vulnerability or targeted the young adult females with the greatest reproductive value to the population. These population-level benefits would be especially pronounced if regional carrying capacity increases via climate change [75,76] and human tolerance for carnivores [77].

We combined these goals and observations to develop a system similar to the kind that operates for people around the world where railways intersect roads and sidewalks. In that context, approaching trains are usually signalled by ringing bells and flashing lights, so we developed a track-mounted, electronic system to provide similar warning signals to wildlife [78]. It works by detecting passing trains via vibration and relaying that information by radio signal to a series of warning devices that are positioned in a mitigation zone approximately 30 s (at posted train speed) from the detector. The warning devices emit bell sounds and flashing lights with components that are small, inexpensive, durable and self-contained [78]. Preliminary results suggest that activation of these devices causes animals near the track to retreat from trains several seconds earlier than animals in the same locations when the device is not activated [79]. Additional work will determine whether track curvature and adjacent topography can predict locations where the sounds of approaching trains are more difficult to detect, potentially contributing to collision hotspots, and suggesting where warning systems could be most helpful [80].

An important aspect of our warning system is its reliance on the principles of associative learning [81], wherein animals are taught to associate a benign conditioned stimulus (the lights and bells) with an unconditioned one (the passage of a train). We exposed wildlife to activated warning stimuli consistently (whenever trains approached), specifically (only for trains), and immediately (within 30 s). Our warning stimuli were not aversive because we assumed the noise, vibration and smell of a passing train is inherently aversive to animals, consistent with our own trackside experience and routine observations of animals fleeing as trains approached. Similar learning principles are assumed to apply to people when roadside animal detection systems are triggered by wildlife to warn drivers of potential collisions. Several reports suggest that these systems reduce collision rates with large animals [82], likely because their association with actual collision risk is robust enough to cause drivers to slow down [83].

To our knowledge, no other system emphasizes learning by wildlife, as opposed to deterrence of wildlife or learning by vehicle drivers, as the basis of mitigating transportation infrastructure, but a few systems use vehicle-triggered deterrents in a similar way. Vehicles themselves could provide warnings prior to their arrival at potential collision sites via sounds emitted from forward-pointing speakers or by reflecting vehicle headlights from roadside posts. Unfortunately, neither method seems to reduce collision rates for cars [84], perhaps because the stimuli are produced inconsistently by only a few passing vehicles. More promising results are emerging for railways. A Polish animal protection device that is triggered by approaching trains emits alarm calls of several local species to frighten animals, which leave the railway an average of 20 s earlier than occurs without the device [85]. A similar device in Japan appears to cause deer to flee the railway as trains approach [86]. Swedish workers are developing a warning system integrated with exclusion fencing that is suspended at designated wildlife crosswalks. When a train approaches a crosswalk, it deploys acoustic and visual stimuli to deter animals temporarily from the area [87].

We posit that learning-based mitigation in the form of warning devices and similar alerting systems could offer economic, logistical, and ecological advantages over the continuous fencing and crossing structures that are favoured for high-traffic roads [12,13] and sometimes advocated for railways [14]. These advantages might be especially pronounced when managers can identify collision hotspots [88] and employ partial fencing [89]. Learning-based mitigation may be most helpful for species, like grizzly bears, with high reproductive skew [90], slow reproductive rates and the capacity to learn about novel predators [91]. Dual benefits for wildlife and people may occur if warning systems are developed in one context, but apply to both, such as systems targeting vulnerable age and sex classes [92,93]. As a group, carnivores are likely to benefit from warning systems due to high behavioural flexibility that is more pronounced in urbanizing areas [94]. Such flexibility is relevant to most ecological contexts [95], but appears to be more advantageous in environments that are variable or changing rapidly [30,96]. Indeed, the residential elk in our study area that ceased migrating to avoid predators and exploit urban food sources (above) were also bolder and more exploratory [97] with less lateralization of limb use, a measure of cognitive flexibility [98].

## 6. Conclusion

In this synthetic paper, we described a ubiquitous, but understudied, form of ecological trap caused by transportation infrastructure: mortality from wildlife–train collisions. This global problem will grow considerably in the decades ahead owing to increasing reliance on railways and higher train speeds, in turn, needed to support simultaneously economic growth and emission targets to limit climate change [99,100]. Our opportunity to study this global problem stemmed from the social context of a charismatic, locally threatened species that appeared to be endangered by a historic, nationally important railway in Canada's first National Park. In this system, we explored the root causes of grizzly bear vulnerability to train strikes with a goal of understanding why they had suddenly increased and to recommend appropriate mitigation that might be applied here and elsewhere. We concluded that multiple, interacting, and changing factors attracted bears to the rail and that mortality sites were not readily explained by spilled agricultural products or any other single cause.

To support behavioural adaptation by wildlife and as an alternative to the high ecological and economic cost of exclusion fencing and crossing structures, we developed a warning device that alerts animals of approaching trains. Such devices could reduce collision rates by targeting hotspots of mortality or locations likely to be used by adult female grizzly bears with high reproductive value who might teach that awareness to their rail-naive offspring. We also support complementary mitigation actions that are already underway in our study area to minimize rail-associated attractants, increase sightlines, and provide alternative travel routes and foraging areas [40]. Interestingly, the past 5 years has witnessed only one human-caused mortality of a grizzly bear in our study area, which stemmed from a collision on a highway. This improvement suggests that past mitigation has been effective, but it also supports the hypothesis that bears needed time to adapt to a landscape that was changing very rapidly as rail-caused mortality began.

We join others in this special issue to show how greater emphasis on animal behaviour, particularly learning, could contribute much to conservation success for populations and ecosystems. Carnivores, with high inherent behavioural flexibility, are especially likely to both need and benefit from learning that enhances coexistence with people [33]. In the context of transportation infrastructure, we think there is particular potential to address limitations of perception [73] and other invisible barriers [101] with the kinds of associative learning that are already widely practiced to increase wariness for both endangered species [91] and species prone to conflict with people [102,103]. As for our study of bears, both problems and solutions are complex and interactive [104]; no silver bullets are likely to emerge and no sacred cows should escape investigation. Future research with more emphasis on animal behaviour, particularly as it relates to learning and flexibility, could rapidly increase the repertoire of techniques available for teaching wild animals to avoid the specific anthropogenic features that create ecological traps.

## Data accessibility

This article has no additional data.
